# Maternal effect senescence and caloric restriction interact to affect fitness through changes in life history timing

**DOI:** 10.1111/1365-2656.14220

**Published:** 2024-11-26

**Authors:** Christina M. Hernández, Silke F. van Daalen, Alyssa Liguori, Michael G. Neubert, Hal Caswell, Kristin E. Gribble

**Affiliations:** ^1^ Department of Ecology and Evolutionary Biology Cornell University Ithaca New York USA; ^2^ Biology Department Woods Hole Oceanographic Institution Woods Hole Massachusetts USA; ^3^ Josephine Bay Paul Center for Comparative Molecular Biology and Evolution Marine Biological Laboratory Woods Hole Massachusetts USA; ^4^ Institute for Biodiversity and Ecosystem Dynamics University of Amsterdam Amsterdam The Netherlands; ^5^ Present address: Wageningen Marine Research IJmuiden The Netherlands; ^6^ Present address: Department of Biology State University of New York at New Paltz New Paltz New York USA

**Keywords:** *Brachionus manjavacas*, life table response experiment, lifespan, matrix population model, reproductive output, rotifer, trade‐offs

## Abstract

1. Environmental factors and individual attributes, and their interactions, impact survival, growth and reproduction of an individual throughout its life. In the clonal rotifer *Brachionus*, low food conditions delay reproduction and extend lifespan. This species also exhibits maternal effect senescence; the offspring of older mothers have lower survival and reproductive output. In this paper, we explored the population consequences of the individual‐level interaction of maternal age and low food availability.
2. We built matrix population models for both ad libitum and low food treatments, in which individuals are classified both by their age and maternal age. Low food conditions reduced population growth rate (Δλ=−0.0574) and shifted the population structure to older maternal ages, but did not detectably impact individual lifetime reproductive output.
3. We analysed hypothetical scenarios in which reduced fertility or survival led to approximately stationary populations that maintained the shape of the difference in demographic rates between the ad libitum and low food treatments. When fertility was reduced, the populations were more evenly distributed across ages and maternal ages, while the lower‐survival models showed an increased concentration of individuals in the youngest ages and maternal ages.
4. Using life table response experiment analyses, we compared populations grown under ad libitum and low food conditions in scenarios representing laboratory conditions, reduced fertility and reduced survival. In the laboratory scenario, the reduction in population growth rate under low food conditions is primarily due to decreased fertility in early life. In the lower‐fertility scenario, contributions from differences in fertility and survival are more similar, and show trade‐offs across both ages and maternal ages. In the lower‐survival scenario, the contributions from decreased fertility in early life again dominate the difference in λ.
5. These results demonstrate that processes that potentially benefit individuals (e.g. lifespan extension) may actually reduce fitness and population growth because of links with other demographic changes (e.g. delayed reproduction). Because the interactions of maternal age and low food availability depend on the population structure, the fitness consequences of an environmental change can only be fully understood through analysis that takes into account the entire life cycle.

## Graphical abstract

With matrix models and individual life histories, this study shows that maternal effect senescence ‐ reduced offspring quality for older mothers ‐ and caloric restriction interact to to affect fitness. In rotifers, low food conditions decreased fitness despite increased lifespan and similar lifetime reproductive output. The decrease was predominantly due to reproductive delays.

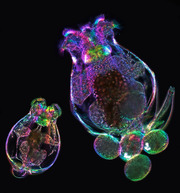

## 1 INTRODUCTION

An individual's demographic performance depends on both intrinsic individual attributes and extrinsic environmental factors that affect its survival and fertility. Some individual attributes, like size or developmental stage, are dynamic and change over the course of an individual's life. Other attributes, such as an individual's genotype or birth weight, are fixed. Fixed and dynamic individual attributes interact with each other and with extrinsic environmental factors (e.g. temperature or the presence of competitors) to determine an individual's life history as well as the fitness of a phenotype. In this paper, we combine individual life history trajectory data with multistate demographic models to demonstrate how an interaction between a dynamic attribute (age), a fixed attribute (maternal age—the age of an individual's mother at the individual's birth), and an environmental factor (food supply) impacts demographic performance in complex ways.

Consider the life history trajectories of the laboratory‐raised rotifers studied by Bock et al. (2019, Figure 1). These rotifers, in the species *Brachionus manjavacas* (Mills et al., 2017), suffer *demographic senescence*—declines in both fecundity and survival with advanced age (Snell, 2014). Demographic senescence is a common (but not universal) characteristic of animal life histories (Jones & Vaupel, 2017). The detrimental effects of demographic senescence on fitness, and theories to explain how demographic senescence might evolve despite those negative effects, have been the subject of many experimental and theoretical studies (for reviews see, e.g. Charlesworth, 2000; Kirkwood & Rose, 1991; Monaghan et al., 2008; Shefferson et al., 2017).

**FIGURE 1:**
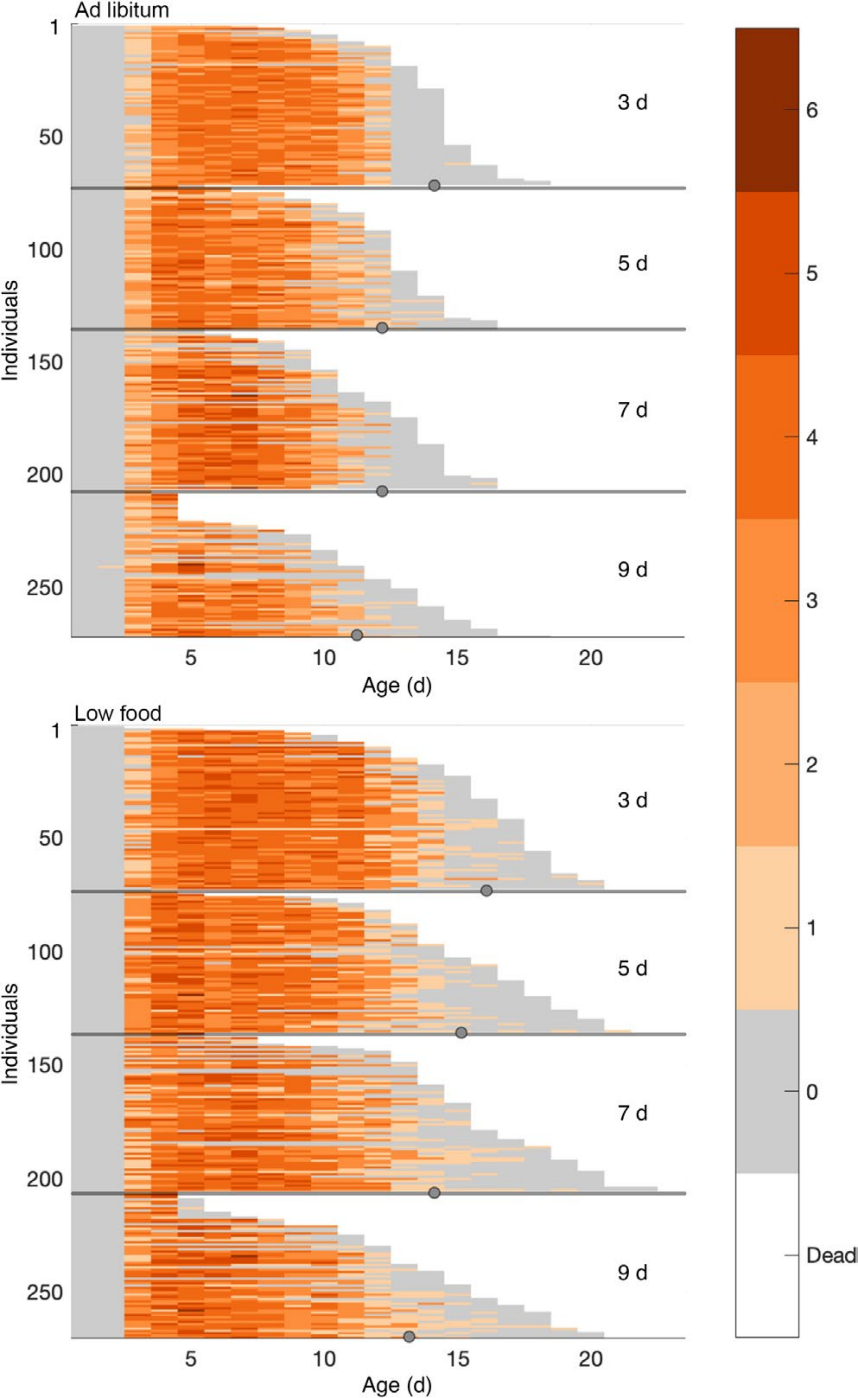
The fate of a rotifer on a given day depends on its age, maternal age, and food supply. In each panel, an individual's survival and reproduction is summarized in a coloured horizontal line. Colours represent the number of offspring produced at a given age. The individuals in the top four panels were raised under ad libitum food conditions; those in bottom four panels were raised in low food conditions. Individuals are grouped by maternal age (3, 5, 7 and 9 days) in each food treatment. Dots mark median lifespan in each group. Data and medians from Bock et al. (2019).

Maternal age can modify the effects of demographic senescence, as demonstrated in *B. manjavacas* (Bock et al., 2019; Figure 1). Individuals born to older mothers have, on average, shorter lives than those born to younger mothers. This phenomenon, known as the “Lansing Effect,” occurs in many animal species (Ivimey‐Cook et al., 2023; Monaghan et al., 2020), including humans (Bell, 1918) and the rotifers studied by Albert Lansing (1947), for whom the effect is named. Maternal age often impacts fertility as well as longevity. For example, Bock et al. (2019) found that both offspring lifespan and lifetime reproductive output decline with increasing maternal age (Figure 1). This kind of reduction in the demographic performance of offspring with advancing maternal age is called ‘maternal effect senescence’ (Moorad & Nussey, 2016). Bock et al. (2019) also found that maternal age affects the timing of reproduction in *B. manjavacas*: advanced maternal age decreased the reproductive period and increased the post‐reproductive period as a percentage of total lifespan.

Shifts in the timing of reproduction, like those exhibited by *B. manjavacas*, can dramatically impact population growth (Caswell & Hastings, 1980; Lewontin, 1965). If a decrease in lifespan is accompanied by changes in the timing of reproduction, the effects of those timing changes may counter the fitness detriments of shorter life due to advanced maternal age. In a contrasting example, Chen et al. (2007) found that two mutant strains of the nematode *Caenorhabditis elegans* with greatly extended lifespan had reduced fitness compared to the reference laboratory strain, because the extended lifespan was accompanied by reduced and delayed reproduction.

Through their complicated effects on survival and reproduction, age and maternal age influence fitness (i.e. population growth rate) in complex ways. It is difficult to understand these impacts, or their evolutionary and ecological ramifications, without a demographic model to link effects on individuals with their population dynamic consequences. Caswell et al. (2018) developed matrix population models that can incorporate two individual attributes. With that approach, we previously developed models for *B. manjavacas* that include survival and fertility rates as functions of age and maternal age as estimated from the experimental data reported by Bock et al. (2019). We used the model to estimate population growth rate, asymptotic population structure and reproductive value, selection gradients and components of variance in lifetime reproductive output (Hernández et al., 2020; van Daalen et al., 2022). We found that maternal effect senescence reduces fitness for *B. manjavacas* and that this decrease arises primarily through reduced fertility, particularly at ages corresponding to peak reproductive output.

The demographic performance of an individual depends not only on individual attributes but also on environmental factors. Among these factors, the availability of energy and nutrients in food can be a primary determinant of performance. Low food levels and starvation are generally detrimental to individuals, leading to population decline (Kirk, 1997; McNamara, 1987); however, chronic caloric restriction without malnutrition is known to extend lifespan in many taxa (Kirkwood & Shanley, 2005; Mattison et al., 2017; McCay et al., 1935; Sutphin & Kaeberlein, 2008; Weindruch et al., 1986). This response to caloric restriction can be beneficial for individuals living in variable environments if the resulting lifespan extension permits reproduction later in life when higher food levels are restored (Holliday, 1989; Kirkwood, 1977; Stearns, 1989).

Gribble, Kaido, et al. (2014) and Bock et al. (2019) demonstrated that chronic caloric restriction (which we will call ‘low food conditions’) extends expected lifespan in *B. manjavacas* but not uniformly (compare Figure 1 ‘Ad libitum’ with Figure 1 ‘Low food’); the magnitude of lifespan extension is proportionately larger in the offspring of older mothers. All else equal, lifespan extension associated with low food conditions would boost fitness. But when, if ever, is all else equal? For *B. manjavacas*, low food conditions also extend the reproductive period and shift fertility later in life. Here again, the changes in fertility and its timing induced by low food depend on maternal age. Bock et al. (2019) found that low food reduced early‐age daily reproductive output (DRO) for young and middle maternal age individuals, but did not decrease DRO for young offspring of older mothers. As a result of its combined effects on lifespan and fertility, low food conditions did not change lifetime reproductive output (LRO) for young and middle maternal ages, but they increased LRO for the offspring of older mothers.

Given the complex impacts of maternal age and low food availability on individual life histories, what are the population‐level effects of their interaction? How do these population‐level effects depend on the demographic context (i.e. population growth rate and population structure)? Bock et al. (2019) suggested that the negative effects of maternal effect senescence offset any fitness benefit from the demographic response to low food conditions, but they did not calculate population growth rate (or other demographic measures of population performance) for lack of an age‐by‐maternal‐age model.

In the following sections, we use experimental data reported by Bock et al. (2019) to estimate a modified version of a matrix population model first developed by Hernández et al. (2020). We estimate the model parameters for offspring raised under either well‐fed (‘ad libitum’) conditions or low food conditions, and use the models to calculate the demographic consequences of the interaction between low food and maternal effect senescence. We also use life table response experiment (LTRE) analyses to decompose the change in population growth rate into its contributions from changes in the rates of survival and reproduction at all combinations of age and maternal age.

The population growth rates we calculate are high under both food treatments; life is good for a laboratory‐raised rotifer, even a hungry one. Such high growth rates, and the young‐skewed age and maternal age structures that accompany them, would likely not persist for long in a natural environment where competition, predation and disease would keep population growth in check. We therefore investigate whether our results would change if either fertility or survival were reduced to bring the population growth rate (λ) close to one.

## 2 THE DATA: INDIVIDUAL LIFE HISTORIES WITH KNOWN MATERNAL AGES AND TWO FEEDING TREATMENTS

The data used in this paper originated in Bock et al. (2019). To study the interaction between maternal age and food supply, Bock et al. (2019) conducted multigenerational life table experiments using the Russian strain of *B. manjavacas* (BmanRUS). The laboratory experimental methods are provided in detail in that paper. In brief, to avoid undefined parental age effects in the experimental populations, Bock et al. (2019) used eggs only from young (3–5 days old) females to establish the great‐grand‐maternal and grand‐maternal generations for the experimental maternal cohort. The maternal generation was established by depositing neonates individually into 1 mL of 15 ppt Instant Ocean and the chlorophyte *Tetraselmis suecica* as food in wells of 24‐well tissue culture plates (*n* = 187). To obtain the offspring cohorts, at maternal ages of 3, 5, 7 and 9 days they isolated one female neonate hatched within the previous 24 h per maternal female. The ages at which to establish the maternal age cohorts were selected to cover the various stages of the reproductive period and lifespan of *B. manjavacas*: early reproduction, maximum daily reproduction, declining reproduction and late reproduction. These offspring were placed individually in wells of 24‐well plates with Instant Ocean and algae according to their food treatment (*n* = 69–72 individuals in each maternal age and feeding treatment cohort). Mothers and the ad libitum fed offspring were provided with 6 × 10^5^ cells/mL *T. suecica*. Individuals in the low food treatment were provided with 10% of the ad libitum food level throughout their lives. Every 24 h, Bock et al. (2019) recorded survival and the number of live offspring for each offspring individual; the female was then transferred to a new well with fresh algae and seawater. Survivorship data were right censored in cases where individuals were lost prior to death. Ethical approval was not required for the rotifer experiments, as invertebrate animals are not subject to ethics regulations.

## 3 THE DEMOGRAPHIC MODEL

Our demographic model uses the general age‐by‐stage structured approach thoroughly described by Caswell et al. (2018), and introduced for the particular setting of rotifers in our previous work (Hernández et al., 2020). For the reader's convenience, we briefly review the model structure here.

If $n_{i,j}\left(t\right)$ is the number of individuals in maternal age class i and age class j on day t, then the population distribution can be concisely summarized in a column vector $\tilde{n}\left(t\right)$, that collects maternal ages within age classes:

(1) $$\tilde{n}\left(t\right)=\left(\begin{array}{c} n_{1,1}\left(t\right)\\ \vdots \\ n_{s,1}\left(t\right)\\ \vdots \\ n_{1,\omega }\left(t\right)\\ \vdots \\ n_{s,\omega }\left(t\right) \end{array}\right).$$

No individual in our laboratory populations reproduced after 19 days of age, so we set both the maximum age (ω) and the maximum maternal age (s) to 19 days in all models.

An individual with maternal age i and age j produces $f_{\mathrm{ij}}$ daughters in a day, and survives to age j+1 with probability $p_{\mathrm{ij}}$. These vital rates are incorporated into a fertility matrix $\tilde{F}$ and a survival matrix $\tilde{U}$. The population projection matrix $\tilde{A}$, which projects the population vector from 1 day to the next, is the sum of $\tilde{F}$ and $\tilde{U}$, and the population dynamics are given by

(2) $$\tilde{n}\left(t+1\right)=\left(\tilde{U}+\tilde{F}\right)\;\tilde{n}\left(t\right)=\tilde{A}\;\tilde{n}\left(t\right).$$

Caswell et al. (2018) describe in detail the construction of $\tilde{U}$ and $\tilde{F}$ for general stage‐by‐age matrix models. The special case where stage is maternal age is described in the Supplementary Information of Hernández et al. (2020). In order to duplicate the results of our analyses, the reader needs:

The matrix $\tilde{U}$. This block matrix takes the form

(3) $$\tilde{U}=\left(\begin{array}{ccccc} 0 & & & & \\ U_{1} & 0 & & & \\ & U_{2} & 0 & & \\ & & \ddots & 0 & \\ & & & U_{\omega -1} & 0 \end{array}\right).$$

The $U_{j}$ are s×s matrices with survival probabilities on the diagonal and zeros elsewhere:

(4) $$U_{j}=\left(\begin{array}{cccc} p_{1,j} & & & \\ & p_{2,j} & & \\ & & \ddots & \\ & & & p_{s,j} \end{array}\right).$$

The matrix $\tilde{F}$. This matrix has a block first row composed of the s×s blocks $F_{j}$:

(5) $$\tilde{F}=\left(\begin{array}{cccc} F_{1} & F_{2} & \cdots & F_{\omega } \end{array}\right).$$

The block $F_{j}$ is a fertility matrix for all females in age class j. Because the offspring of a mother of age j have maternal age j, the matrix $F_{j}$ contains zeros everywhere except in the *j*‐th row, where the vector $f_{j}=\left(f_{1,j},f_{2,j},\ldots ,f_{s,j}\right)$ appears.

A population described by model (2) will eventually grow at the rate λ (the largest eigenvalue of the projection matrix $\tilde{A}$) and converge to a stable stage‐by‐age structure $\tilde{w}$ (the right eigenvector corresponding to λ). We treat the intrinsic growth rate λ associated with a phenotype as a measure of the fitness of that phenotype (e.g. Hamilton, 1966; Lande, 1982; Metz et al., 1992).

## 4 MODELLING AND ANALYSIS

### 4.1 Constructing matrix population models for ad libitum and caloric restriction treatments

The laboratory experiments provided measures of daily fertility and survival for individuals with maternal ages 3, 5, 7 and 9 days. To build a matrix population model, we need estimates of fertility and survival parameters for every possible maternal age. Hernández et al. (2020) fit parametric functions of age and maternal age for both fertility and survival of individuals in the ad libitum feeding treatment. To avoid the restrictions of using a parametric form for the low food treatment data, we took a non‐parametric approach in the current study, described in the next several paragraphs. To be consistent in our model construction and use of the laboratory data, we re‐fit the ad libitum data using the same non‐parametric approach. This flexible non‐parametric approach may be suitable for a wide range of other taxa and settings.

**FIGURE 2:**
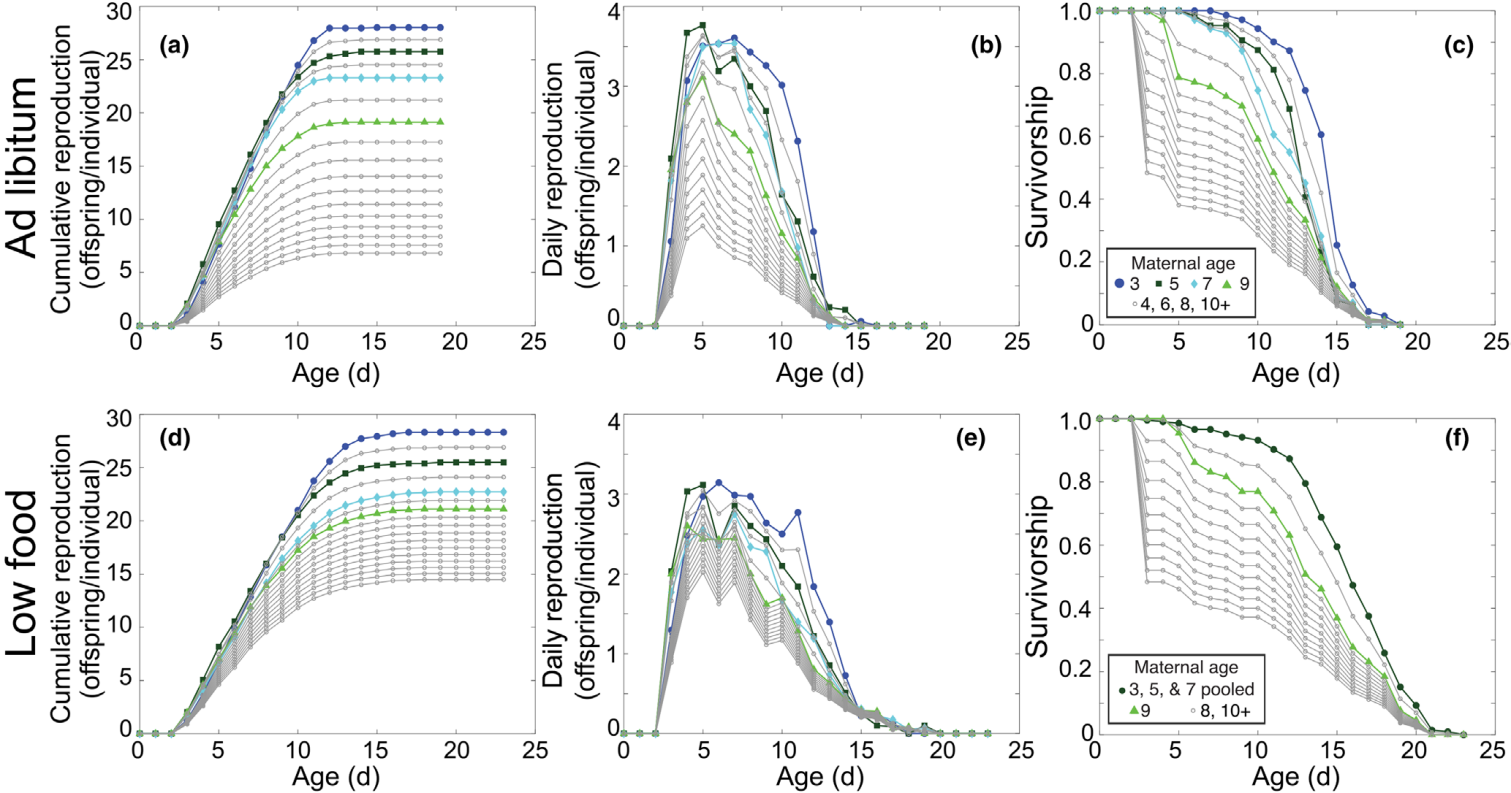
Population data and non‐parametric model fits for reproduction and survivorship for rotifers of different maternal ages. For both ad libitum (top row) and low food (bottom row) laboratory populations, we interpolated and extrapolated reproductive output using cumulative reproduction curves (a, d). The daily reproduction rates (b, e) used for parameterizing the matrices were calculated as the daily differences from the cumulative reproduction curves. For survivorship (c, f), we interpolated for maternal ages 4, 6 and 8 days but did not extrapolate outside of the observed maternal ages. In the case of survivorship for low food treatment (f), observations from maternal ages 3, 5 and 7 days were pooled. Data are plotted as coloured dots and lines, interpolated curves and extrapolated curves are plotted as grey lines with open circles. The legend shown in panel (c) applies to all panels except for (f).

Our non‐parametric model for change in fertility with age and maternal age was based on interpolation and extrapolation of the cumulative fertility curves. While the daily fertility rates fluctuate (Figure 2b,e), cumulative fertility curves must be monotonically increasing. We calculated the mean values of the daily fertility rates for all maages that were measured in the laboratory, then took the cumulative sum of these to generate cumulative fertility curves (Figure 2a,d). We linearly interpolated the cumulative curves for maternal ages 4, 6 and 8 days. To extrapolate to maternal ages 10 through 19 days, we calculated the percentage decrease in total cumulative fertility between maternal ages 8 and 9 days total cumulative fertility. For each successive day, we decreased the entire cumulative fertility curve by this same percentage, up to maternal age 19 days (Figure 2a,d). The interpolated and extrapolated cumulative curves were then converted back into daily curves by taking the daily difference (see Figure 2b,e). The fertility of individuals with maternal age 1 or 2 days was set to that of the maternal age 3 days individuals.

We used a similar approach to design a non‐parametric model for the effect of maternal age on survival. For the ad libitum feeding treatment, we used the observed curves for maternal ages 3, 5, 7 and 9 days. We linearly interpolated to calculate the curves for maternal ages 4, 6 and 8 days. Because survivorship must be monotonically decreasing, we calculated a multiplier for extrapolation, similar to the percentage decrease used to extrapolate the cumulative fertility curves. We also maintained an initial survivorship plateau at 100% survival until age 3 days across all maternal age groups, such that the extrapolation begins at age 4 days. In the case of survivorship, we found a multiplier for the maternal age 8 days curve that minimized the difference between a predicted maternal age 9 days curve and the observed maternal age 9 days curve. This multiplier was then successively applied to produce the survivorship curves for maternal ages 10–19 days (Figure 2c).

Under low food treatment, the survivorship curves for maternal ages 3–7 days do not significantly differ (Figure S2c). Therefore, we pooled the survivorship data from individuals with maternal ages 3, 5 and 7 days and applied that mean curve to maternal ages 1 through 7 days in our matrix model for low food conditions. We calculated the survivorship curve for maternal age 8 days as a linear interpolation between the pooled 3/5/7 days curve and the 9 days survivorship curve. We then extrapolated curves for maternal ages 10–19 days following the same procedure as for the ad libitum data (Figure 2f).

The daily survivorship and fertility curves for ad libitum feeding and low food conditions (Figure 2) were then used to parameterize an age‐ and maternal‐age‐classified matrix population model assuming a birth‐flow process with a time step of 1 day.

**FIGURE 3:**
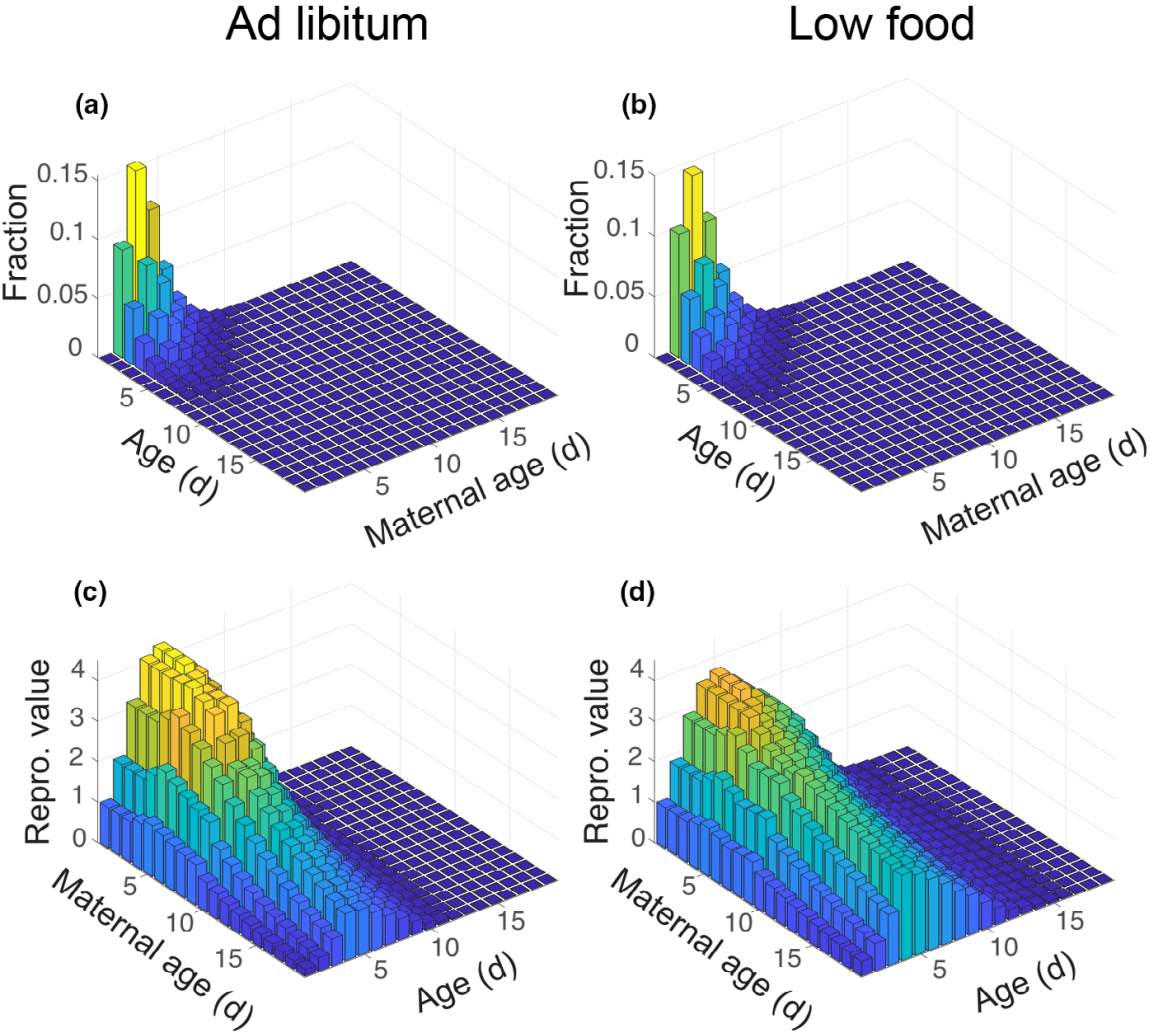
Stable population distribution and reproductive value by age and maternal age for ad libitum and low food feeding treatments. The height of each bar, as well as its colour, represents the portion of the stable population comprised of that age and maternal age (a, b) or the reproductive value of individuals in that age and material age class (c, d). For visualization of the treatment effects, panels (a and b) share a *z*‐scale and corresponding colour scale, as do panels (c and d). Note that the age and maternal age axes are flipped between the first and second rows.

**FIGURE 4:**
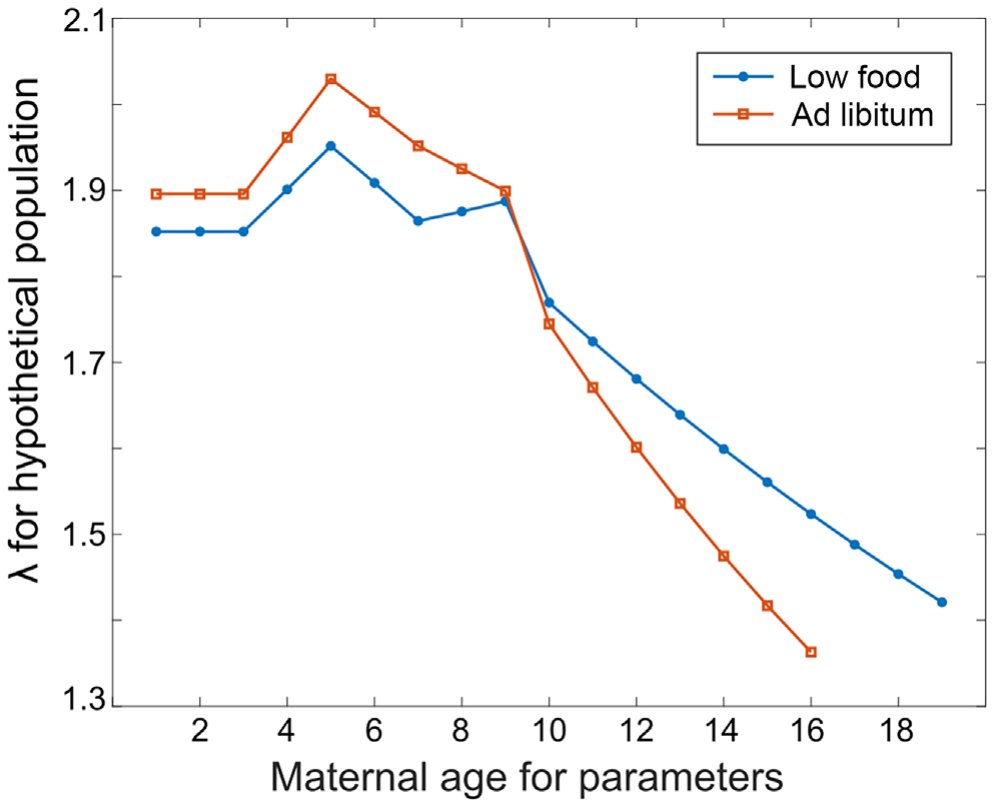
Population growth rates for hypothetical populations with survival and fertility rates corresponding to the possible maternal ages and food treatments. Each point represents the population growth rate (λ) for a population where all individuals have the survival and fertility rates corresponding to a given maternal age and feeding treatment.

**FIGURE 5:**
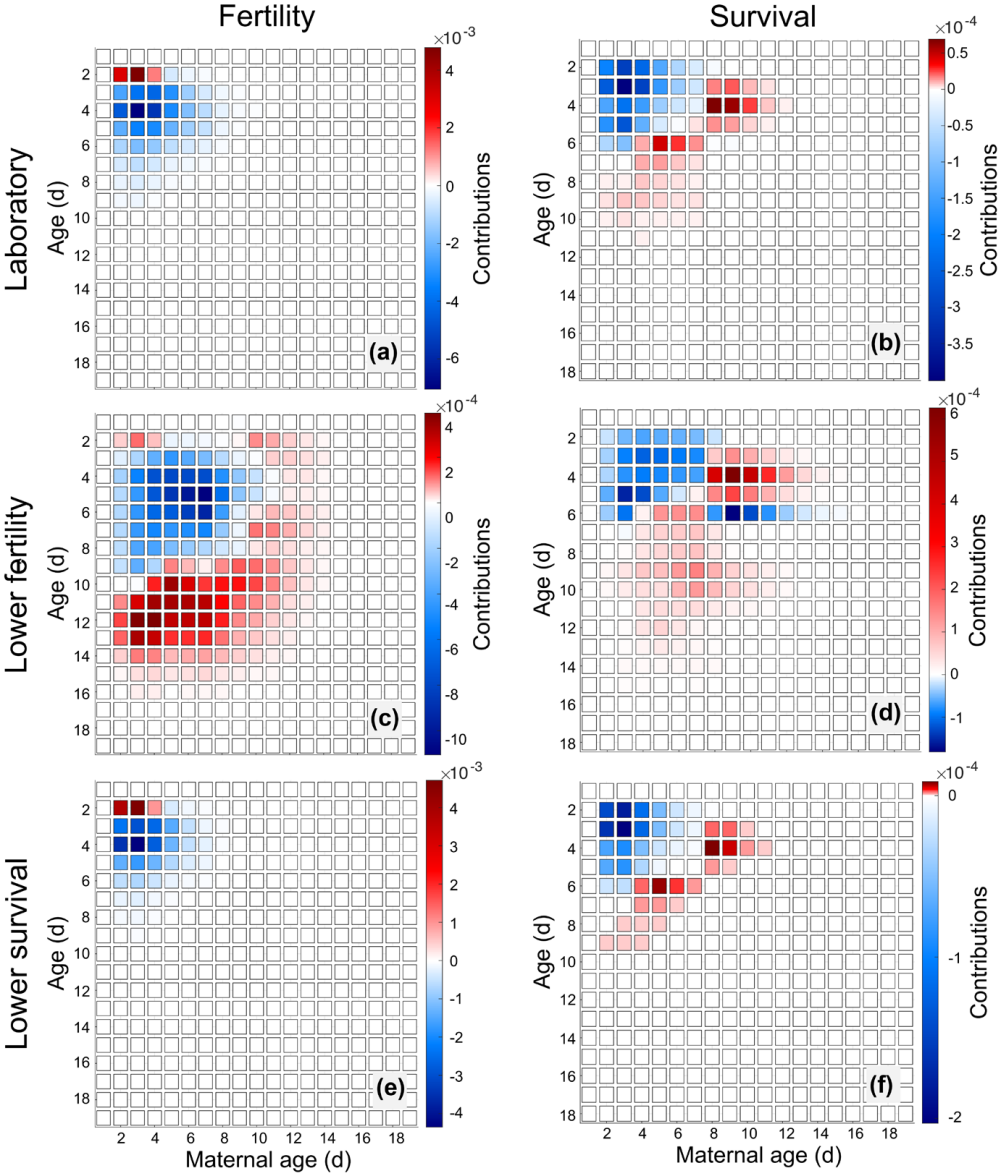
LTRE comparing ad libitum (AL) and low food (LF) treatments. We show panels for the contributions from fertility (left column) and survival (right column) from the laboratory (first row), low‐fertility (second row), and low‐survival scenarios (bottom row). In all cases, the LTRE is performed for $\Delta \lambda =\lambda^{\left(LF\right)}-\lambda^{\left(AL\right)}<0$. Note that the colour scale is unique in each panel, with red colours always indicating a positive contribution and blue colours a negative contribution to λ. Figure S5 shows this same figure with symmetric colour axes.

We tested the sensitivity of our results to the choice of survival model parameterization by comparing our results with those resulting from a model with no extrapolation to older maternal ages. In this alternative, conservative model, individuals with maternal age 10 days and older are assigned the observed survivorship curve from maternal age 9 days. The changes to our results (i.e. Figures 3, 4, 5) were imperceptible.

### 4.2 Demographic effects of caloric restriction

We used permutation tests to investigate whether the population growth rate (λ), the net reproductive rate ($R_{0}$) and the stable population structure were significantly different under ad libitum feeding versus low food conditions. For these permutation tests, we created counterfactual data sets where individual‐level data (i.e. the vector of daily observations of survival and reproductive output) were randomly assigned to either ad libitum or low food conditions. In other words, individuals were ‘shuffled’ between feeding treatments, while retaining their maternal age class and sample sizes for each maternal‐age‐by‐feeding‐treatment combination. For each of these shuffled data sets, we re‐calculated the population projection matrices ($\tilde{A}$) for ‘ad libitum’ and ‘low food’ treatments using the same non‐parametric fitting methods described above. We then calculated the λ, net reproductive rate ($R_{0}$) and stable population distribution for both projection matrices.

We performed 4000 permutations to generate a null distribution for the absolute value of the difference in λ, absolute value of the difference in $R_{0}$, and the 1‐norm of the difference in the stable population structure. These null distributions were then used to evaluate the significance level of the observed differences in λ, $R_{0}$ and the stable population structure under ad libitum feeding and low food conditions. We used the absolute value of the difference in λ and $R_{0}$ because we are primarily interested in how often extreme differences would be possible by chance, and less interested in directionality of the difference.

The laboratory rotifers had high population growth rates (λ), indicative of the populations nearly doubling every day. Similar to our previous results (Hernández et al., 2020), we estimated λ to be 1.9363 for the ad libitum population. Caloric restriction decreased λ to 1.8789 (Δλ=−0.0574). Although this difference in λ may seem small, it would have substantial ecological implications given that daily population growth rates are exponential. For example, over a typical growing season of 90 days, the ad libitum population would be 15 times larger than the low food population ceteris paribus.

As expected with such high population growth rates, the stable population structure was dominated by individuals with young age and young maternal age in both food treatments (Figure 3). Permutation tests showed that the effect of feeding treatment was highly significant for population growth rate (p<0.00025) and stable population structure (p=0.0012; Figure S3 and Table S1).

Low food conditions delayed reproduction, but did not markedly decrease overall reproduction (*R*_0_ = 21.8576 for ad libitum treatment and 20.8998 for low food treatment) or the number of days that individuals were reproductively active (Figure 2). The permutation test showed that the effect on $R_{0}$ was not statistically significant compared to the null distribution (p=0.244). We can also visualize the shifting of reproductive activity to a later and wider peak by looking at the reproductive value of individuals with different ages and maternal ages (Figure 3c,d). Under low food conditions, the peak reproductive value was lower, but individuals maintained higher reproductive value at older ages and older maternal age relative to the ad libitum treatment. In fact, the effect of low food conditions on the shape of the reproductive value surface is more apparent along the maternal age axis, with the oldest maternal age classes maintaining a peak reproductive value above 2 for the low food treatment.

We calculated λ for a series of hypothetical populations in which all individuals experience the rates of a single maternal age group. We found that the curves of population growth rate for the different feeding treatments cross at approximately maternal age 9 days, demonstrating the interaction of caloric restriction and maternal effect senescence (Figure 4). For hypothetical populations corresponding to the parameters for individuals with young maternal ages, the ad libitum treatment led to a higher λ value than the low food treatment. Both feeding treatments yielded a peak λ value at maternal age 5 days, but that peak fell off more steeply for ad libitum feeding than for low food. By maternal age 10 days, the λ values for low food conditions were higher than those for ad libitum conditions. Therefore, caloric restriction increased the contribution of older maternal age classes to population growth, similar to the individual‐level results of extending reproductive value and offspring production to older ages. At the population level, caloric restriction mitigated the impact of maternal effect senescence on λ.

### 4.3 Analysis of stationary populations

The laboratory population had extremely high population growth rates and a population structure that was heavily concentrated in young ages and young maternal ages. To see how low food conditions would interact with maternal effect senescence in stationary populations (with λ≈1) we reduced the entries in the population projection matrices for both the ad libitum and low food treatments by reducing either fertility or survival (following Hernández et al., 2020). In the low fertility and low survival scenarios, we chose to scale the ad libitum and low food models by the same constant, because this preserved the age‐specific patterns of changes in demographic rates due to maternal effect senescence and food treatments. The low fertility scenario could arise through competition for resources, while the low survival scenario might result from an ecosystem with high predation pressure.

For the low fertility scenario, we divided all fertility entries in each projection matrix by the net reproductive rate ($R_{0}$) from the ad libitum treatment. This yielded λ=1 for the ad libitum treatment, and λ=0.9929 for the low food treatment. For the low survival scenario, we solved for the constant multiplier of the survival matrix ($\tilde{U}$) that would give λ=1 for the ad libitum population; this constant was 0.4023. We then scaled the survival matrices for both feeding treatments by this constant, yielding λ=1 for the ad libitum treatment, and λ=0.9762 for the low food treatment.

As we saw in our previous work (Hernández et al., 2020), compared with the laboratory population, the low‐fertility scenario exhibited a population structure that was more evenly distributed across age and maternal age classes, while the low‐survival scenario exhibited a higher proportion of the population in young ages and young maternal ages (Figure S4). The transformation from high‐growth to low‐fertility and low‐survival scenarios preserved the difference between ad libitum and low food treatments, so that the stable population structure under low food always has a slightly flatter distribution than that under ad libitum conditions, with more individuals from older ages and older maternal ages represented in the stable structure.

### 4.4 Life table response experiments comparing ad libitum and low food treatments

We used fixed design Life Table Response Experiment (LTRE; Caswell, 1989, 2001, Chapt. 10) analyses to decompose the difference in population growth rate (λ, given by the largest eigenvalue of the projection matrix) into contributions from the differences in the vital rates (i.e. entries in the matrices):

(6) $${\left.\lambda^{\left(\mathrm{LF}\right)}-\lambda^{\left(\mathrm{AL}\right)}\approx \sum_{i,j}\left(a_{\mathrm{ij}}^{\left(\mathrm{LF}\right)}-a_{\mathrm{ij}}^{\left(\mathrm{AL}\right)}\right)\frac{\partial \lambda }{\partial a_{\mathrm{ij}}}\right|}_{\bar{A}},$$

where $\bar{A}$ is the mean of the two population projection matrices being compared,

(7) $$\bar{A}=\frac{1}{2}\left({\tilde{A}}^{\left(\mathrm{LF}\right)}+{\tilde{A}}^{\left(\mathrm{AL}\right)}\right).$$

Note that in these equations we use “LF” to indicate the low food treatment and “AL” to indicate the ad libitum treatment.

We performed LTRE to compare the ad libitum and low food treatments. We did this under three settings: (1) the high‐growth laboratory scenario, (2) the hypothetical low‐fertility scenario and (3) the hypothetical low‐survival scenario.

The LTRE comparison between the laboratory treatments showed that decreased fertility at young maternal ages accounted for most of the decrease in λ under low food conditions (Figure 5). The positive contributions from fertility at age 2 days for maternal ages 2, 3 and 4 days (Figure 5a) are due to the birth‐flow census design, because some of the individuals observed at census age 3 days were allocated to age 2 days. Individuals with age 3 days and maternal age 3 days had slightly higher reproductive output under low food conditions than under ad libitum conditions (Figure 2), but at age 4 days the ad libitum individuals have much higher reproductive output. Therefore, the LTRE shows positive contributions from differences in fertility at age 2 days but negative contributions at age 3 days. Overall, the sum of contributions from fertility was −0.0542, or 94.4% of the difference in λ. The contributions from survival were an order of magnitude smaller than those from fertility (5.6% of Δλ), but they extend into older ages and maternal ages and showed a more obvious trade‐off pattern (Figure 5b). At young ages (2–6 days) and young maternal ages (2–7 days), the contributions from survival were negative. However, there were positive contributions from survival at slightly older ages and maternal ages. Interestingly, these positive contributions occurred at ages 6–11 days for maternal ages 2–8 days, and at ages 3–5 days for maternal ages 8–11 days. So, at younger maternal ages, which dominated the population, the positive effects of low food did not manifest until older ages. Meanwhile, at older maternal ages, the positive effects of low food on survival were realized before/at the age of peak reproduction.

Under the low fertility scenario, the contributions from fertility and survival were of the same order of magnitude and exhibited trade‐offs across ages and maternal ages as well as between survival and fertility. For fertility, there were negative contributions at young ages (3–8 days) and positive contributions at older ages (10–14 days) for maternal ages 2–9 days (Figure 5c). At older maternal ages, contributions from fertility were small but positive. The negative contribution of the depressed peak reproductive output under low food conditions was compensated by the extension of reproduction to older ages. The contributions from survival likewise compensated across ages and maternal ages, with negative contributions at young ages (2–6 days) and young maternal ages (2–7 days), and positive contributions at young ages for middle maternal ages and middle ages for young maternal ages (Figure 5d). The line of blue squares that is visible within the red part of Figure 5d is due to the steeper drop‐off in survival at age 6 for individuals from older maternal ages in the ad libitum rather than low food treatment. There was also compensation between fertility and survival: the total contribution from differences in fertility was 1.56 times as large as Δλ, while the total contribution from differences in survival was 0.52 times as large as Δλ and had the opposite sign.^1^

The LTRE results from the low‐survival scenario look like a more extreme version of the results from the laboratory scenario, in keeping with the effects of decreased survival on the stable population structure. The contributions from fertility were an order of magnitude larger than the contributions from survival, were nearly all negative (Figure 5e), and were concentrated in young ages and maternal ages (2–6 days). Meanwhile, survival contributions were negative for the youngest ages and maternal ages (2–5 days) and positive for *either* slightly older maternal ages or slightly older ages (6–10 days, Figure 5f).

## 5 DISCUSSION

Our results demonstrate that low food and maternal effect senescence interact to alter the vital rates of individuals and highlight the way in which life history timing determines how effects on individuals scale up to impact the population. Although low food led to potential individual benefits in the form of lifespan extension without reducing lifetime reproductive output, the delayed onset of reproduction and lower daily fecundity decreased λ such that within 90 days (a summer season), a population growing at the rate under ad libitum food would be 15 times larger than one growing at the rate under low food. Because of this interaction between caloric restriction and maternal age, the fitness consequences of caloric restriction depend on maternal age, and we observed trade‐offs between survival and fertility across ages and across maternal ages. This interaction between an individual attribute (maternal age) and an environmental factor (food level) highlights the difference between the potential fitness effects of single life history traits and the net effects of interacting traits. Therefore, a fuller understanding of how fitness is impacted by an environmental factor, such as food availability, requires that researchers also account for intrinsic differences, such as maternal age.

Laboratory conditions are not representative of long‐term ‘natural conditions,’ as evidenced by the extremely high growth rate observed in the laboratory populations. The laboratory data used here came from experiments in which rotifers were cultured individually in well plates with constant food and stable temperatures, and without predators or disease. To simulate a more natural situation, we performed post‐hoc manipulations of the model to explore the interaction of maternal effect senescence and feeding treatments when population size is approximately stationary. These simulations revealed that the population‐level effects of caloric restriction were strongly influenced by population structure, since the low‐fertility and low‐survival scenarios had similar growth rates but strikingly different patterns of the effect of caloric restriction on the population growth rate. When extrapolating laboratory data to an ecological context, it is important to understand how the laboratory conditions influence the conclusions drawn from experimental results.

Our LTRE results reveal interesting patterns suggestive of life history compensation in the face of low food conditions. These are particularly evident in the low‐fertility scenario, in which the populations had much flatter population distributions than in the laboratory setting and low‐survival scenario (Figure S4). The presence of more individuals with older ages and older maternal ages increased the strength of life history compensation that is visible but weak in the laboratory and low‐survival scenarios (Figure 5).

Low food conditions shifted the maximum daily reproduction to older ages (Figure 5c), leading to negative contributions to population growth rate at younger ages and positive contributions at older ages; these at least partially cancel out. We also saw compensation across maternal ages in the response of survivorship to low food conditions (Figure 5d): at young maternal ages, the lower survival under low food conditions decreased population growth, but at older maternal ages, the survivorship under low food conditions was higher than that under ad libitum conditions. In other words, the responses of survival and reproduction to low food conditions weaken the negative impact of maternal effect senescence, which in turn lessens the decrease in fitness due to low food conditions. These low‐fertility scenario results underscore the importance of aging processes that might not be observed in laboratory data because of high population growth rates and populations skewed towards young individuals.

To understand individual and population responses to caloric restriction requires an understanding of the interaction between reproduction and longevity, a longstanding question in evolutionary biology. In our rotifer system, caloric restriction is potentially beneficial for individuals in that it extends lifespan, but it also shifts the timing of fertility. It has been theorized that lifespan extension would be selected for in environments where timescales of food limitation are typically on the timescales of individual lifespans (Holliday, 1989; Kirkwood & Shanley, 2005; Shanley & Kirkwood, 2000). Delaying reproduction during food shortage periods and extending lifespan may allow individuals to take advantage of food in the future and to maximize lifetime reproductive output (Harrison & Archer, 1989). In this study, rotifers were exposed to low food conditions without restoring high food levels later in life. Comparing phenotypes in constant environments representing high and low resource availability may under‐ or overestimate a genotype's fitness in a variable environment. This is particularly relevant for rotifers, which inhabit highly ephemeral aquatic environments and exhibit a wide range of responses to changes in food availability across species (Kirk, 1997; Weithoff, 2007). An interesting avenue for future research will be to investigate the effects of interactions between maternal effect senescence and different magnitudes and timescales of variability in food availability on population dynamics and fitness. The physiological mechanisms causing maternal age effects and regulating the response to caloric restriction are areas of active and ongoing investigation.

Our model assumes that there are no trans‐generational or inter‐generational effects of maternal diet. The mothers of individuals used to generate the life history data used here were fed ad libitum (Bock et al., 2019). In a wide range of organisms, maternal diet affects offspring phenotype (Bonduriansky & Day, 2009; Mousseau & Fox, 1998), including body size (Donelson et al., 2009), development (Hafer et al., 2011; Zizzari et al., 2016), lifespan (Ivimey‐Cook et al., 2021), fecundity (Harvey & Orbidans, 2011), and physiology (Saastamoinen et al., 2013). In *Brachionus manjavacas*, offspring that were fed ad libitum with mothers that were reared under caloric restriction had longer lifespans and higher fecundity than those with well‐fed mothers (Gribble, Jarvis, et al., 2014). Maternal diet can also interact with offspring diet to affect fitness (Bonduriansky & Head, 2007; Deas et al., 2019). Maternal diet effects can be a result of adaptation, in which mothers modify the phenotypes of offspring to anticipate environmental change, which should be predictable from mothers' experiences, to maximize their fitness (Burgess & Marshall, 2014). Conversely, maternal diet effects could be a side effect of physiological constraints (Marshall & Uller, 2007). Future work investigating the impacts of food availability on life history evolution should consider environmental variability both within and across generations (Stearns, 1989).

Multi‐state structured population models can be applied to investigate other systems and stressors. We have shown the utility of the approach for understanding the interaction of caloric restriction and maternal effect senescence, but it could also be used to explore the interaction of maternal age effects with other environmental factors. Food limitation is one type of stressor that is important in variable environments (Deas et al., 2019; Saastamoinen et al., 2013; Sutphin & Kaeberlein, 2008; Weithoff, 2007). Temperature during development has been shown to have long‐lasting effects on individuals (e.g. birds, Uehling et al., 2020; fruit flies, Hercus & Hoffmann, 2000; mammals, Descamps et al., 2008; fish, Scott & Johnston, 2012). In aquatic environments, other abiotic factors that can strongly affect vital rates include salinity, pH, oxygen availability, and toxins or pollutants (Crain et al., 2008; Todgham & Stillman, 2013). Biotic factors such as disease and pathogens (Clark et al., 2017), shifts in population density (Plaistow & Benton, 2009) and predation (Gilbert & McPeek, 2013) have also been studied in combination with maternal age effects. Where there are life history data as well as information on both maternal age and exposure to stressors (Fox & Dingle, 1994; Goos et al., 2019; Hercus & Hoffmann, 2000; Lord et al., 2021; Plaistow & Benton, 2009), our approach will be valuable for exploring and disentangling the interaction between these intrinsic and extrinsic factors.

This investigation of the interplay of caloric restriction, age and maternal age demonstrates a fundamental tension between potential benefits to an individual and the resulting fitness of a phenotype. While individuals may experience lifespan extension under caloric restriction, the resulting shift to later reproduction causes a lower fitness. The fitness of a given phenotype is a measure that integrates across the life cycle, incorporating both the magnitude and the timing of reproduction. Our matrix population modelling approach enables this type of life history integration and underscores the sensitivity of fitness to the timing of individual life history events. Ultimately, our results show remarkable compensation across ages and maternal ages in the face of low food conditions, suggesting that rotifer life history responses to the combination of intrinsic and extrinsic factors lead to smaller effects on fitness than if only one of these factors were operating.

## AUTHOR CONTRIBUTIONS

All authors conceived of the project. Kristin E. Gribble provided data. Christina M. Hernandez and Michael G. Neubert performed analyses and prepared figures. All authors contributed to interpretation of results. Christina M. Hernandez and Alyssa Liguori wrote the first draft, and all authors contributed to revising the text.

## CONFLICT OF INTEREST STATEMENT

The authors declare no conflict of interest.

## Supporting information


**Table S1.** Permutation test results for *λ*, *R*_0_, and the stable population structure.
**Figure S1.** Observed and pooled survivorship data for the low food treatment, and how it compares with ad libitum treatment survivorship.
**Figure S2.** Confidence bands for survivorship and fertility curves for *ad libitum* and low food treatments.
**Figure S3.** Significance testing for the effect of food treatment on (a) *λ*, (b) *R*_0_, and (c) the stable population structure.
**Figure S4.** Stable population structures for *ad libitum* and low food treatments for low fertility and low survival scenarios.
**Figure S5.** LTRE results plotted with symmetric color axes in each panel.

## Data Availability

The data that support the findings of this study are openly available at https://zenodo.org/doi/10.5281/zenodo.11125048 (Hernández et al., 2024).

## Footnotes

^1^
The total contributions do not exactly equal Δλ because the LTRE analysis used here is an approximation.
