# Supplementary material for: Maternal effect senescence and caloric restriction interact to affect fitness through changes in life history timing
Source: J Anim Ecol. 2024 Nov 26;94(1):99–111. doi: 10.1111/1365-2656.14220 (PMC11730777; doi:10.1111/1365-2656.14220)
Supplement: Supplementary file 1 — Table S1. Permutation test results for λ, R 0, and the stable population structure. Figure S1. Observed and pooled survivorship data for the low food treatment, and how it compares with ad libitum treatment survivorship. Figure S2. Confidence bands for survivorship and fertility curves for ad libitum and low food treatments. Figure S3. Significance testing for the effect of food treatment on (a) λ, (b) R 0, and (c) the stable population structure. Figure S4. Stable population structures for ad libitum and low food treatments for low fertility and low survival scenarios. Figure S5. LTRE results plotted with symmetric color axes in each panel. [file JANE-94-99-s001.pdf]

Supplemental Figures and Tables for Hernandez *et al.* "Maternal effect senescence and caloric restriction interact to affect fitness through changes in life history timing"

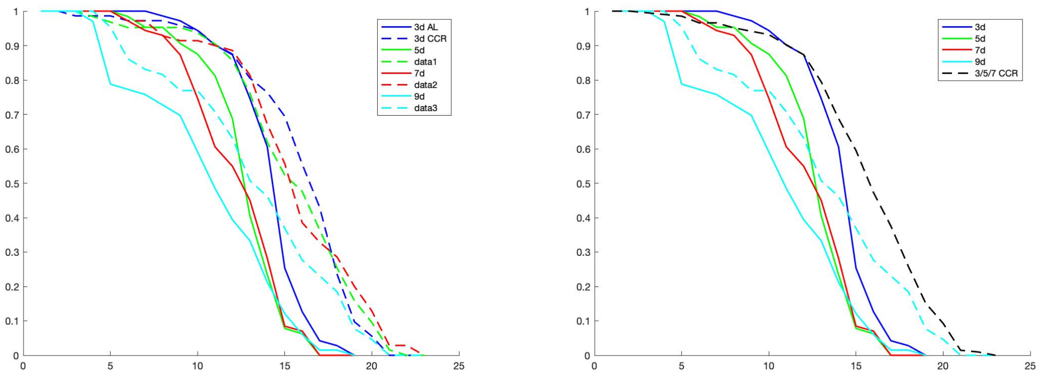

(a) (b)

Figure S1: Observed and pooled survivorship data for the low food treatment, and how it compares with *ad libitum* treatment survivorship. In (a), we plot the observed survivorship curves for low food treatment and *ad libitum* feeding. In (b), survivorship curves from the low food treatment are shown as pooled for maternal ages 3, 5, and 7 d.

Table S1: Permutation test results for λ, R_0, and the stable population structure.

**Metric**	**Observed value**	**Proportion of null distribution smaller than observed value**
$\|\Delta\lambda\|$	0.0572	100%
$\|\Delta R_0\|$	0.5797	53.5%
$\|\|\mathbf{w}_{AL} - \mathbf{w}_{LF}\|\|_1$	0.0770	99.88%

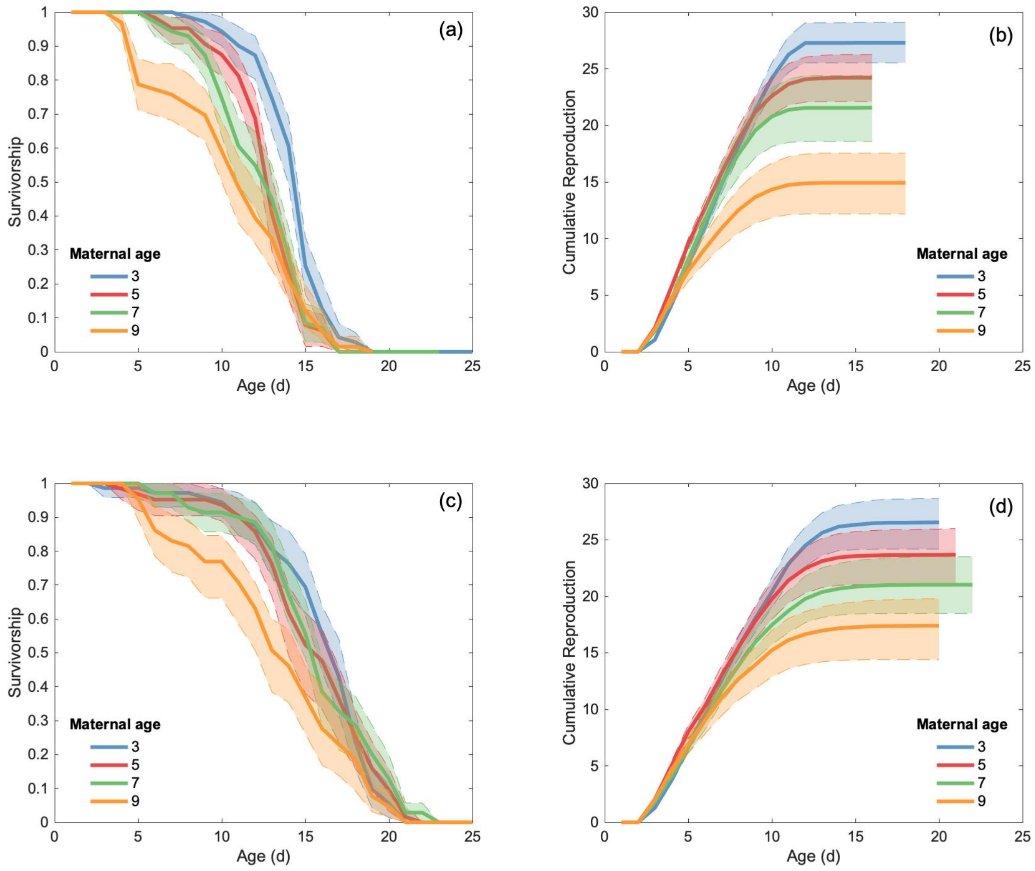

Figure S2: Confidence bands for survivorship and fertility curves for *ad libitum* and low food treatments. Survivorship is shown in panels a and c, while fertility is shown in panels b and d. The top row (a and c) correspond to ad libitum feeding and the bottom row (c and d) correspond to the low food treatment.

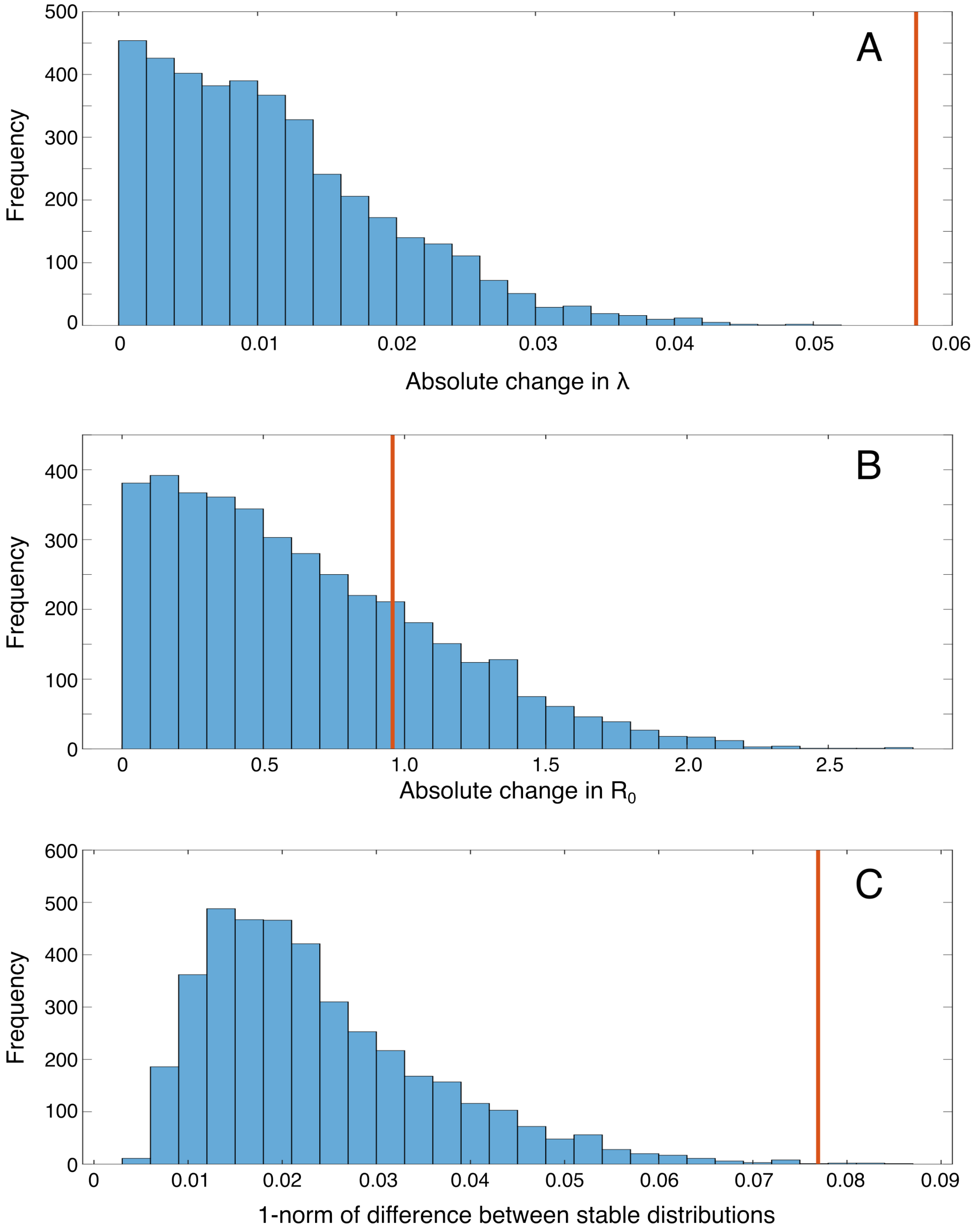

Figure S3: Significance testing for the effect of food treatment on (a) λ, (b) R_0, and (c) the stable population structure. We calculated the absolute value of the difference in λ or R_0 between the *ad libitum* and low food feeding treatments. For the stable population structure, we calculated the 1-norm of the difference between the stable population structure vectors. In each panel, the bars represent a histogram of values resulting from 4000 permutations of the data, and the vertical line shows the observed value calculated for the laboratory populations.

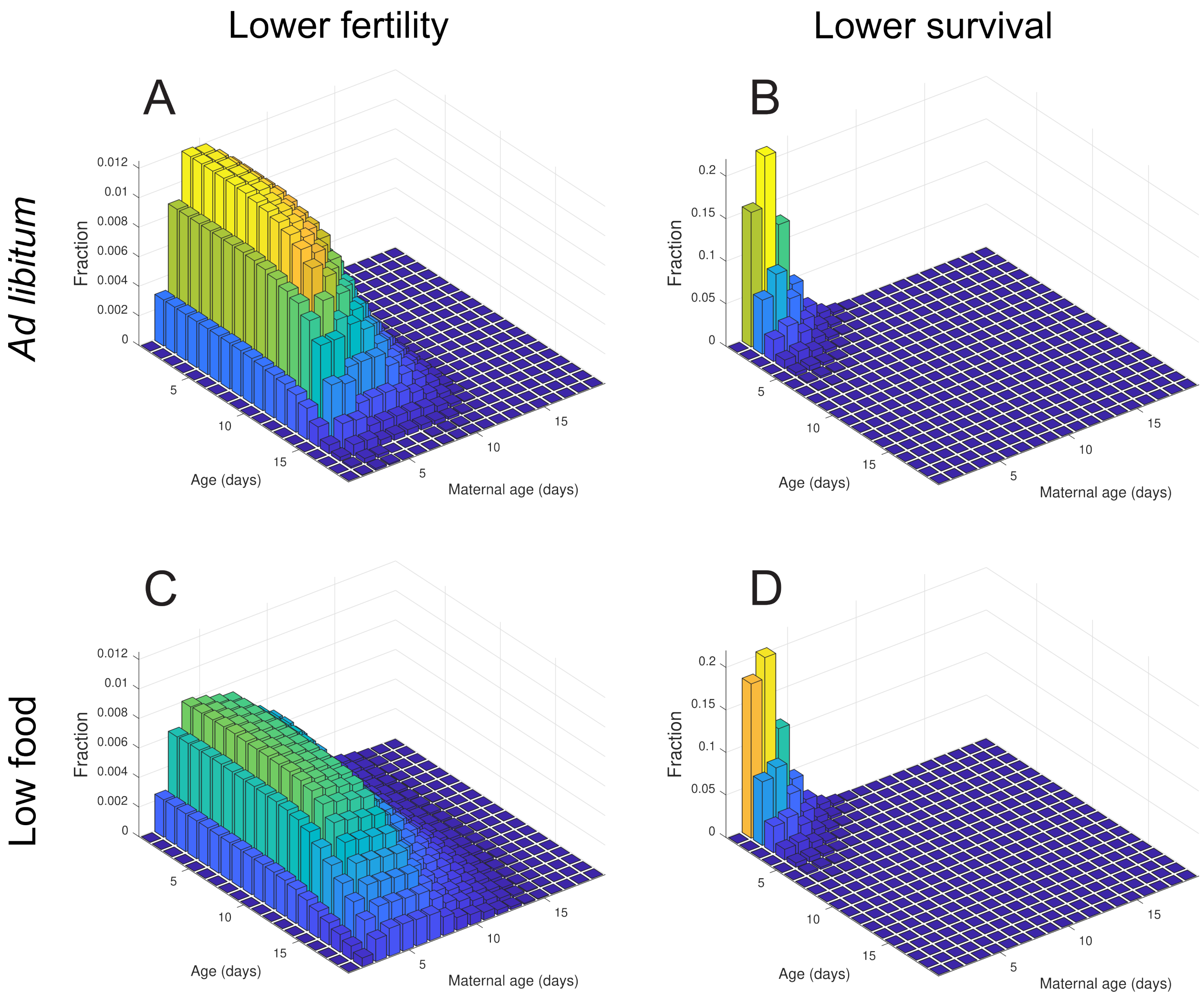

Figure S4: Stable population structures for *ad libitum* and low food for low fertility and low survival scenarios. The upper row (A and B) corresponds to *ad libitum* feeding conditions, while the lower row (C and D) corresponds to low food conditions. The left column (A and C) corresponds to the lower-fertility scenario, while the right column (B and D) corresponds to the lower-survival scenario.

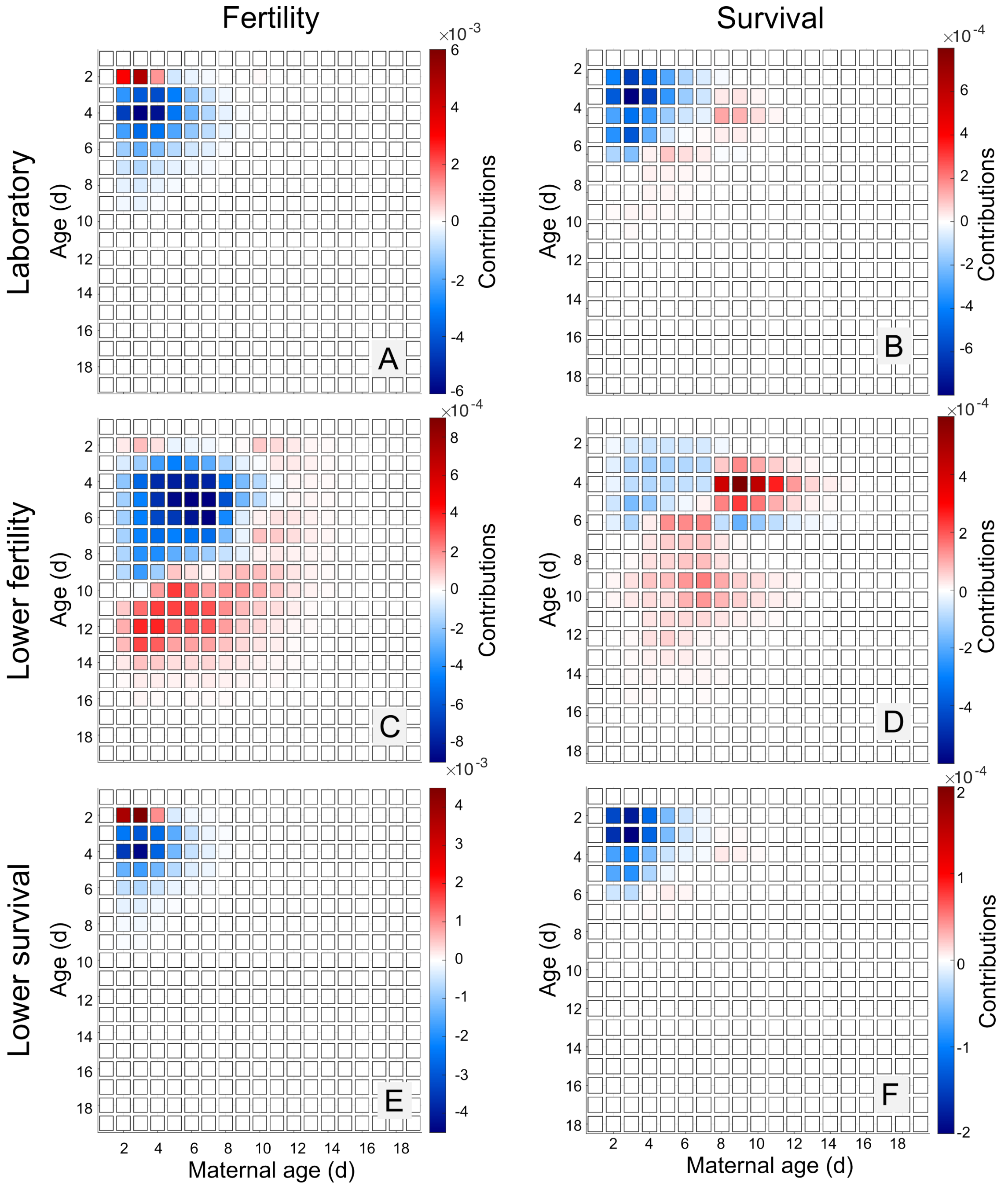

Figure S5: LTRE results plotted with symmetric color axes in each panel. We show panels for the contributions from fertility (left column) and survival (right column) from the laboratory (first row), low-fertility (second row), and low-survival scenarios (bottom row). In all cases, the LTRE is performed for $\Delta\lambda = \lambda(LF) - \lambda(AL) < 0$. Note that the color scale is unique in each panel, with red colors always indicating a positive contribution and blue colors a negative contribution to λ. This figure is the same as Figure 5 in the main text, but all color axes are symmetric: within each panel, blue and red colors of the same intensity indicate the same absolute value.
